# Comprehensive and Region-Specific Retinal Health Assessment Using Phasor Analysis of Multispectral Images and Machine Learning

**DOI:** 10.3390/s26031021

**Published:** 2026-02-04

**Authors:** Armin Eskandarinasab, Laura Rey-Barroso, Francisco J. Burgos-Fernández, Meritxell Vilaseca

**Affiliations:** Center for Sensors, Instruments and Systems Development (CD6), Universitat Politècnica de Catalunya (UPC), Rambla Sant Nebridi 10, 08222 Terrassa, Spain

**Keywords:** phasor analysis, multispectral imaging, retinal disease classification, machine learning

## Abstract

This study examines the efficacy of phasor analysis in distinguishing between healthy and diseased retinas using multispectral imaging data together with machine learning approaches. Our results demonstrate that phasor analysis of multispectral images surpasses average reflectance values in classification performance, serving as an effective dimensionality reduction technique to extract essential features, with the first harmonic yielding optimal results when paired with Z-score normalization. To compare the effectiveness of multispectral images with that of a conventional color fundus camera, we extracted three spectral bands corresponding to the red, green, and blue regions and combined them to create RGB-like images, which were then subjected to the same analysis. Our study found that phasor analysis of multispectral images provided more accurate classification results than phasor analysis of RGB-like images. An examination of different regions of interest showed that using the entire retina yields the best classification performance, likely due to the advanced stage of the diseases, which had progressed to affect the entire fundus. Our findings suggest that phasor analysis of multispectral images and machine learning are a powerful tools for retinal disease classification.

## 1. Introduction

Retinography offers a high-resolution, non-invasive visualization of the eye fundus, enabling ophthalmologists to meticulously examine critical structures, including the blood vessels, optic disk, and macula. As a result, fundus imaging has emerged as a vital diagnostic tool, playing a crucial role in the detection of abnormalities, diagnosis of retinal diseases, and development of tailored treatment strategies.

Fundus cameras typically use color sensors with three broad RGB bands to capture image data. The detection of diseases using RGB fundus cameras has been extensively investigated in the literature using various image processing methods, and Machine Learning (ML) and Deep Learning (DL) techniques. With advancements in ML and DL, fundus imaging has become an essential tool for detecting abnormalities, diagnosing retinal diseases, and guiding personalized treatment plans. For example, in a study [1] the authors analyzed 249,620 fundus images labeled for 39 different retinal issues, using a DL-based method to classify images related to disease and conditions. Similar studies have used image processing, besides ML, and DL methods, to detect various diseases using RGB fundus images [2,3,4,5]. Despite these successes, RGB fundus images inherently compress rich spectral information into three broad bands.

In contrast, multispectral and hyperspectral imaging offers a more advanced approach by capturing information across a wider range of wavelengths, including those beyond the visible (VIS) spectrum. This can potentially reveal subtle changes and critical details. These images can capture both spectral and spatial details of the retina simultaneously [6]. These spectral fundus eye images have been used to help detect various ocular diseases such as Age-related Macular Degeneration (AMD) [7,8], Diabetic Retinopathy (DR) [9,10], performing retinal oximetry [11,12,13], and also other applications dealing with the detection of neurological disorders such as detection of Alzheimer’s disease [14,15,16,17].

In a recent study [18], researchers employed a spectrometer to capture spectral data of different regions of the retina (five measurements), which was then used to reconstruct high-fidelity spectral reflectance (380–780 nm) from standard RGB fundus images. By deriving a transformation matrix from a 24-color checkerboard calibration, the system enabled hyperspectral reconstruction. For that, 3256 RGB fundus images from 137 patients were captured and transformed into hyperspectral images for further analysis. Their findings demonstrated that hyperspectral images outperform RGB images in accurately identifying mild cognitive impairment and dementia versus normal cases. Importantly, even when hyperspectral information is synthetically reconstructed from RGB images, it leads to improved diagnostic performance.

The availability of RGB fundus images far surpasses that of multispectral fundus images, primarily due to the rarity of multispectral fundus cameras in clinical centers and eye clinics. Consequently, research on multispectral fundus images is limited compared to RGB fundus images. To address this, studies have focused on making these cameras more accessible to the clinical community and improving their utility in diagnostic applications, such as developing cameras that can capture fast multispectral images [19] or reducing the cost of production [20].

Multispectral cameras capture a large amount of data corresponding to multiple bands, often more than 10 spanning from infrared to VIS and even ultraviolet, which presents both opportunities and challenges. On the one hand, it provides a rich source of information valuable for diagnosis and analysis. On the other hand, the vast amount of data can be difficult to preprocess, may lead to redundancy, and can be challenging for both ML algorithms and humans to process and interpret.

To address this challenge, phasor analysis might serve as an effective dimensionality reduction technique, extracting essential features and rendering the data more manageable. This approach, well-established in fluorescent lifetime imaging microscopy [21], has recently been applied to represent spectral data [22,23], demonstrating its utility in analyzing high-dimensional data. The power of phasor analysis lies in its ability to represent complex spectral signal combinations as linear ones, simplifying tasks such as classification and potentially yielding higher accuracy. Notably, its application to retinal multispectral imaging remains a largely underexplored area.

The transformation of multispectral data into a compact phasor representation significantly alters the learning problem, shifting the focus from high-dimensional feature discovery to classification in a low-dimensional, structured feature space. This change in approach reduces the reliance on DL architectures, which are typically well-suited for learning complex hierarchical representations from large volumes of raw data. However, when dealing with compact phasor features, particularly in situations where the available dataset size is limited, classical ML methods can offer competitive performance while mitigating the risk of overfitting and enhancing interpretability. This is consistent with findings from comparative studies, which have shown that classical ML approaches can perform strongly, especially in classes with lower number of cases and examples relative to deep models [24].

This study aims to assess the efficacy of phasor analysis as a compact spectral representation for multispectral fundus imaging in retinal disease classification. The analysis is conducted on a dataset of multispectral fundus images comprising both healthy and diseased retinas, for which the limited number of available samples motivates the use of traditional ML approaches. Our analysis compares the performance of phasor-based features extracted from multispectral and RGB-like images, as well as untransformed reflectance data, using ML classifiers. Furthermore, we perform a region-wise analysis of the retina (examining the vessels, optic disk, macula, and background) to identify the most informative regions for distinguishing between healthy and diseased retinas.

## 2. Materials and Methods

### 2.1. Experimental System

**Figure 1:**
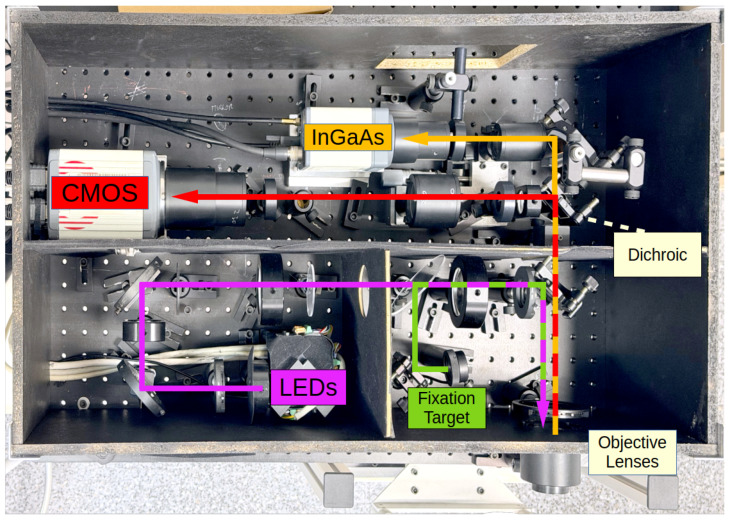
Multispectral fundus camera system and components of the optical camera.

The multispectral images were acquired using a custom-built, area-scanning multispectral fundus camera (Figure 1), explained in [19,25]. This camera features a set of LEDs, each emitting light at a distinct peak wavelength, capturing images using a high-resolution CMOS sensor (Orca Flash 4.0, Hamamatsu Photonics, Hamamatsu, Japan) and an InGaAs camera (C12741-03, Hamamatsu Photonics, Hamamatsu, Japan) covering 15 spectral bands across a range of 416 to 1213 nm.

The high-resolution CMOS sensor (2048×2048 pixels, pixel size 6.5 μm, and 16-bit depth) captures 12 images illuminated using LEDs, with specific peak wavelengths at 416, 450, 471, 494, 524, 595, 598, 624, 660, 732, 865, and 955 nm. In contrast, the lower-resolution InGaAs camera (640×512 pixels, pixel size 20 μm, and 14-bit depth) captures images in 3 bands of 1025, 1096, and 1213 nm. Following post-processing and cropping the black margins surrounding the retina results in a final image resolution of 1757×1757 pixels for the CMOS camera and 386×386 pixels for the InGaAs camera, respectively, from their original sensor sizes.

The system sequentially illuminates the retina in each spectral band. The duration of each LED illumination is 11 ms, during which the CMOS camera captures an image. The system then waits for an additional 11 ms to allow the sensor to save and send the image to the computer. Consequently, the total time required to capture a multispectral image with 12 bands is 264 ms. Meanwhile, the incorporation of three Near-Infrared (NIR) bands, captured using an InGaAs camera, increases the total acquisition time to 612 ms for each study case.

The VIS bands exhibit a significantly higher spatial resolution compared to the NIR bands (more than 5 times). Furthermore, the InGaAs camera’s dark current noise and longer acquisition time for NIR bands make the NIR images of lower quality and besides that, increase susceptibility to artifacts from involuntary eye movements. Furthermore, the higher water absorption of the biological tissue in the NIR range compared to VIS wavelengths results in a lower Signal-to-Noise Ratio (SNR) in the NIR images. This, combined with the former factors, led to the decision to exclude the three NIR images from the study.

To mitigate the effects of involuntary eye movements, such as saccades and micro-movements, three strategies are considered. First, the high-speed sequential recording capability of the device helped to minimize inter-band displacement and motion artifacts; that is, only 264 ms to acquire a complete multispectral sequence when using only the CMOS camera. Second, a fixation target was used to assist patients in maintaining a stable gaze, and the multispectral imaging sequence was captured several times to ensure that high-quality images with minimal artifacts were obtained. Third, the multispectral imaging sequence was set to start acquiring NIR bands to avoid initial pupil constriction and to prevent motion artifacts due to the patient being dazzled from the beginning. In cases where significant motion artifacts or misalignments were still present, the affected image sequences were excluded from the dataset to ensure the quality and reliability of the results.

### 2.2. Dataset and Image Processing

We analyzed a dataset of multispectral images corresponding to 60 diseased retinas and 73 healthy controls, totaling 133 cases. The diseased retinas in this study mainly comprised cases of AMD, drusen, Posterior Vitreous Detachment (PVD), epiretinal membrane, and pathological myopia. Other conditions included diabetic retinopathy, glaucoma, retinal detachment, diabetic macular edema, anterior uveitis, bullous retinoschisis, neovascularization, and macular hole. Table 1 provides more information about each condition; Those with less than 3 cases have been grouped together as “Other conditions”.

The images were sourced from two ophthalmic centers: the Instituto de Microcirugia Ocular (IMO– Miranza Group) in Barcelona, Spain, and the Vision University Center (CUV) of the Universitat Politècnica de Catalunya in Terrassa, Spain. All multispectral images were independently reviewed and annotated by two expert ophthalmologists (C.M. and J.L.G.) to ensure reliable classification into healthy and diseased categories. To complement this quality-controlled labeling process and provide a clearer understanding of the study population, Table 2 summarizes the refractive status of all included cases. In this table, we report the range, mean, and Standard Deviation (STD) for each parameter to offer a more detailed quantitative overview of the dataset.

To obtain accurate pixel-wise reflectance values from the raw multispectral images, a calibration procedure was employed, based on reference white and dark current corrections. This approach, established in previous studies on retinal multispectral imaging [19,25], involved acquiring images of a calibrated reference white target and the corresponding dark current image (captured without illumination and with no eye present) using the same exposure settings as the fundus images. By applying this procedure to each spectral band, we were able to compensate for wavelength-dependent variations in LED output power, camera sensitivity, and background noise, thereby ensuring accurate reflectance measurements. The reflectance at each pixel (i,j) and wavelength $\lambda_{n}$ was computed as follows:

(1) $$Refl_{\lambda_{n}}\left(i,j\right)=\frac{DL_{\lambda_{n}}\left(i,j\right)-DL_{\lambda_{n}}^{Dark}\left(i,j\right)}{DL_{\lambda_{n}}^{White}\left(i,j\right)-DL_{\lambda_{n}}^{Dark}\left(i,j\right)}\times Refl_{\lambda_{n}}^{ref}$$

where $DL_{\lambda_{n}}\left(i,j\right)$ is the digital level of the fundus image, $DL_{\lambda_{n}}^{Dark}\left(i,j\right)$ is the dark current image, $DL_{\lambda_{n}}^{White}\left(i,j\right)$ is the reference white image, and $Refl_{\lambda_{n}}^{ref}$ is the calibrated reflectance of the reference white provided by the manufacturer.

### 2.3. Phasor Analysis

Phasor analysis was performed on each pixel in the spectral cube of a sample, represented as a three-dimensional array (*x*, *y*, λ). The spectrum at each pixel was expressed as a complex number with real and imaginary components ($g_{x,y}+is_{x,y}$) and processed using a discrete Fourier transform [22]. The real and imaginary parts of the phasor values were defined as follows:

(2) $$g_{x,y}\left(k\right)=\frac{\sum_{\lambda_{0}}^{\lambda_{n}}I_{x,y}\left(\lambda \right)\cos\left(\frac{2\pi k\lambda }{2}\right)\Delta \lambda }{\sum_{\lambda_{0}}^{\lambda_{n}}I_{x,y}\left(\lambda \right)\Delta \lambda },s_{x,y}\left(k\right)=\frac{\sum_{\lambda_{0}}^{\lambda_{n}}I_{x,y}\left(\lambda \right)\sin\left(\frac{2\pi k\lambda }{2}\right)\Delta \lambda }{\sum_{\lambda_{0}}^{\lambda_{n}}I_{x,y}\left(\lambda \right)\Delta \lambda }$$

where $\lambda_{0}$ and $\lambda_{n}$ represent the wavelengths of the first and last bands of the multispectral image, respectively. *n* denotes the number of spectral channels in the spectral cube, Δλ represents the bandwidth of a single channel, and *k* refers to the harmonic number.

**Figure 2:**
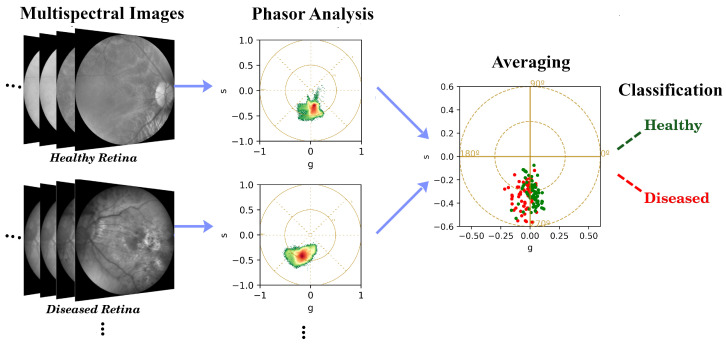
Representation of the phasor analysis process and the classification. The multispectral images of each retina are converted into phasor values, where each phasor corresponds to a single pixel in the original image. These phasor values are then averaged to obtain a single representative point for each eye (entire retina). The averaged values are used as input features for ML classification algorithms to distinguish between healthy and diseased cases.

To summarize the phasor values for each image, we calculated the average $g_{x,y}$ and $s_{x,y}$ values across all pixels corresponding to one entire retina, resulting in a single vector $\left(g_{avg},s_{avg}\right)$, referred to as the averaged representation (Figure 2). This is to minimize pixel-level variability and enhance feature robustness. This averaging process preserves the global spectral, phasor characteristics, while effectively reducing the impact of local noise and heterogeneity, which can compromise subsequent statistical tests and classification. Also for further analysis, the STD of the phasor values was computed to provide a measure of the variability inside one retina [26].

**Figure 3:**
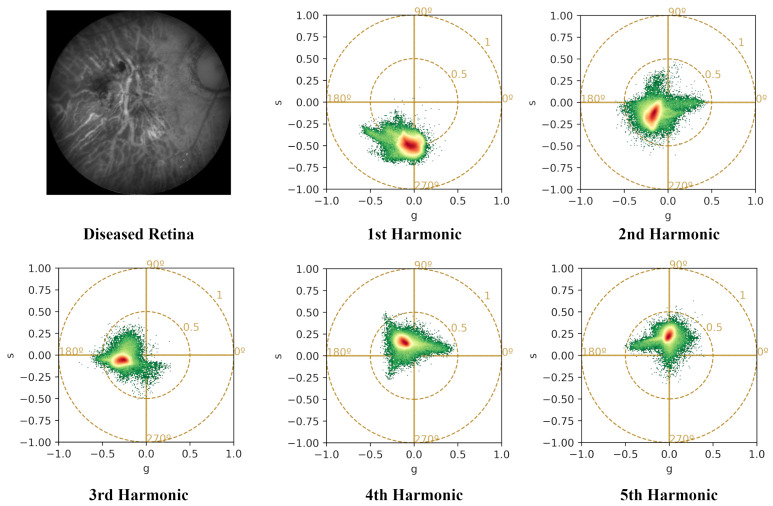
Phasor analysis retrieved from the 12-band multispectral image of a diseased retina. From top left to bottom right: a reflectance image for 624 nm, taken out from the original multispectral image, and 1st to 5th harmonics which are calculated taking into account all 12 bands. Higher harmonics correspond to higher frequencies linked to the smoothness of the spectral curve of eac pixel.

**Figure 4:**
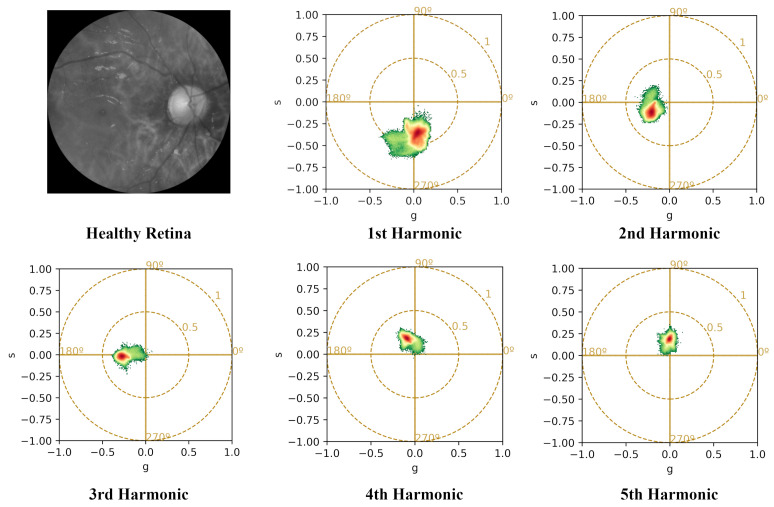
Phasor analysis retrieved from the 12-band multispectral image of a healthy retina. From top left to bottom right: a reflectance image for 624 nm, taken out from the original multispectral image, and 1st to 5th harmonics which are calculated taking into account all 12 bands. Higher harmonics correspond to higher frequencies linked to the smoothness of the spectral curve of each pixel.

The Fourier transform, implemented via Fast Fourier Transform (FFT), yields 12 harmonics (equal to the number of spectral bands). However, since the harmonics start from k=0 (representing the image average), the phasor values are produced for k=1 to 11, resulting in real and imaginary parts. As an example, the difference between harmonics is illustrated for a diseased retina in Figure 3 and a healthy retina in Figure 4.

The normalization of reflectance values in multispectral images corresponding to different bands has a significant impact on phasor values, which in turn can affect the final classification results. To standardize the values, we applied a Z-score normalization using the following equation:

(3) $$Z=\frac{X-\mu }{\sigma }$$

where *X* is the original reflectance value in a specific pixel of a spectral band, μ is the mean of the reflectance values across that band, and σ is the STD of the reflectances in that band. This process was applied band-wise, allowing us to standardize the reflectances before calculating the phasor values. Notably, this normalization method ensures that the values of one band do not affect the others. Besides that, the normalization accentuate the subtle changes at particular wavelengths, which can have a more significant impact on the classification outcome.

In addition to the average phasor values, we also considered the STD of the phasor distribution as a statistical descriptor for each retina. It measures the degree of variability in phasor values, providing information that complements the average values. Since pathological changes in retinal tissue can alter optical properties and subsequently impact spectral characteristics, measures of variability, like STD, was included among the evaluated features to assess whether phasor variability contributes to discrimination between healthy and diseased retinas.

### 2.4. ML Classifiers

Given the small size of our dataset, we opted for traditional ML classifiers over DL methods. We evaluated several algorithms, including Nearest Centroid (NC) [27], Gaussian Naive Bayes (GNB) [28], Support Vector Machine (SVM) [29], and the ν-Support Vector Classifier (ν-SVC) [30]. These classifiers work as follows: NC assigns new instances to the class with the closest centroid; GNB applies Bayes’ theorem, assuming Gaussian-distributed and independent features; SVM finds the optimal hyperplane separating classes; and ν-SVC, a variant of SVM, uses a parameter ν to control the number of support vectors and margin. Each algorithm offers a distinct approach to classification, providing flexibility in tackling diverse datasets and problem requirements.

The classifier training process involves splitting our dataset into two parts: 70% for training and 30% for testing. We used the averaged phasor values $\left(g_{avg},s_{avg}\right)$ as input features and trained the classifiers to distinguish between healthy and diseased cases. To investigate the impact of STD, we also calculated this statistical factor for the phasor values and used it as an additional feature, resulting in a three-feature input set: $\left(g_{avg},s_{avg},STD\right)$.

To facilitate a direct comparison between multispectral and RGB representations, we simulated an RGB-like image from the multispectral data by combining three bands centered at 471, 595, and 732 nm, which correspond to the blue, green, and red channels, respectively. Notably, the resulting RGB-like images therefore represent three-band reflectance images. Afterwards, we applied phasor analysis to this RGB-like image using the same processing pipeline as for the multispectral images, resulting in a phasor-based RGB representation (Phasor-RGB). This approach enabled a controlled assessment of phasor analysis performance across different spectral (color) resolutions, while keeping the underlying image content and feature extraction strategy constant.

In addition to phasor-based features, we also extracted reflectance-based baseline features to provide a reference for simple spectral averaging. For reflectance-based features, we computed the average reflectance value for each spectral band and used these values as input to the classifiers. Specifically, multispectral images were represented by a 12-dimensional feature vector, denoted as Avg-MSI, which comprised the band-wise average reflectance values. In contrast, RGB-like images were represented by a 3-dimensional feature vector, denoted as Avg-RGB.

In this study, we evaluated the performance of the classifiers using six distinct metrics: Overall Accuracy (OA), also referred to as Accuracy; Balanced Accuracy (BA), also known as Average Accuracy; Specificity; Sensitivity; Precision; and F1 Score. These metrics, which are detailed in [31], provide a comprehensive assessment of the classifier’s performance.

The performance of the classifiers was evaluated using a repeated random train-test split approach. The dataset was randomly divided into 70% training and 30% test sets over 10 independent repetitions. In each repetition, the classifier was trained on the training set and evaluated on the corresponding test set. The final performance aforementioned metrics, were computed as the mean values across the 10 repetitions.

To ensure consistency and reproducibility, a fixed random seed was used to generate the same 10 random partitions for all experiments. Although stratified sampling was not explicitly applied, the dataset’s approximate balance between healthy and diseased cases (with a consistent class distribution across random splits) mitigated potential biases.

### 2.5. Statistical Test

To gain a deeper understanding of the impact of the chosen parameters across different scenarios, we also conducted a statistical analysis of the features. Specifically, we performed statistical tests to compare the distributions of phasor features between healthy and diseased retinas. However, commonly used statistical tests, such as the *t*-test, typically assume that the feature of interest (or dependent variable) is a scalar value for each subject. To address this issue, we considered Multivariate Analysis of Variance (MANOVA), with a significance level of p<0.05. The multivariate test employed was Wilks’ Lambda test. This test quantifies the ratio of within-group variance to total variance [32].

To ensure a consistent comparison in cases where each retina is represented by averaged phasor values, we utilized two dependent variables, namely *g* and *s*, within a MANOVA framework. On the other hand, to guarantee alignment across different image representations, we also applied MANOVA to the averaged reflectance values obtained from both RGB-like and multispectral images. Specifically, we considered the average reflectance values (Avg-RGB and Avg-MSI) across the spectral bands as the dependent variables. For these representations, the number of dependent variables matched the number of spectral bands: 3 for RGB-like images and 12 for multispectral images.

### 2.6. Region-Based Analysis

Each fundus image was initially analyzed as a complete retinal image. To further investigate whether region-specific analysis can improve disease detection, the full retinal image was then divided into anatomically distinct regions, including the vessels, optic disk, macula, and background. Each Region of Interest (ROI) was manually segmented, and masks were created to isolate them. We then applied phasor analysis and classification to distinguish between healthy and diseased cases for each region separately. Furthermore, for each region, we created a corresponding dataset containing only the diseases relevant to that region, effectively using a subset of the overall dataset.

**Figure 5:**
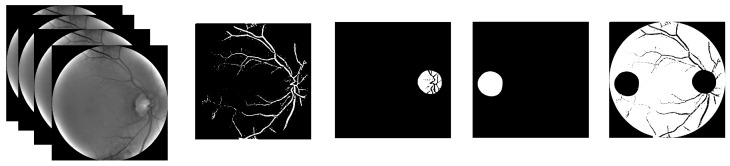
Masks for a representative retina: (left to right) original multispectral image, and manually segmented masks for the vessels, optic disk, macula, and background.

Image masks were employed to isolate areas of interest. Four distinct regions were manually segmented: the optic disk, macula, vessels, and background. An example of a multispectral image and its corresponding masks is provided in Figure 5.

## 3. Results

Initially, a comparison of various classification algorithms was conducted to identify the most effective classifier for further analysis. This evaluation was performed using the classification results obtained from applying average phasor values, extracted from multispectral images of the entire retina, without any normalization. The classification performances are compared for the 4 ML classifiers: NC, GNB, SVM, and ν-SVC. The comparative results using the first harmonic are presented in Table 3.

In the next step, we conducted a comparative analysis of different harmonics to determine the most effective one for classification. We evaluated the performance of various harmonics using the ν-SVC classifier, which had previously shown the highest accuracy (Table 4). The results indicate that the first harmonic consistently outperformed higher-order harmonics. This behavior is consistent with the smooth nature of spectral reflectance curves in biological tissues. Higher-order harmonics, which correspond to higher-frequency components, are more sensitive to noise and local fluctuations and therefore contribute less to robust classification.

To quantitatively assess group differences between healthy and diseased retinas across harmonic orders, we performed a statistical comparison using MANOVA (Table 5). Based on the statistical analysis, the MANOVA results indicate that all harmonics exhibit strong significant differences (*p* < 0.05) between healthy and diseased groups. Given the superior classification performance observed for the first harmonic and its statistically significant separation under MANOVA, this harmonic was selected for subsequent analyses.

**Figure 6:**
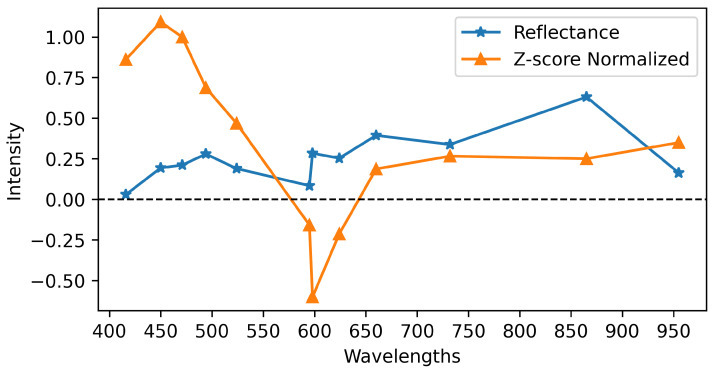
Spectrum changes after Z-score normalization.

Furthermore, we investigated the impact of Z-score normalization and the inclusion of STD on the classification performance. To apply Z-score normalization, we first normalized the multispectral image data, which concentrated most pixels within the range of [−3,3]. However, some pixels exhibited significantly large values, potentially skewing the mean. To address this, we removed the top and bottom 20th percentiles of the data, effectively trimming outliers and yielding a more representative distribution. The modified data were then used to calculate the phasor values. To illustrate the effect of normalization on the spectrum, Figure 6 shows the average spectra of a 200×200 pixel region in an multispectral image before and after Z-score normalization. The normalized spectrum exhibits negative values and displays more pronounced changes compared to the original signal.

By standardizing each spectral band to zero mean and unit variance, Z-score normalization reduces the dominance of absolute reflectance differences and enhances relative, wavelength-specific deviations. This allows subtle spectral changes at particular wavelengths, potentially associated with pathological alterations, to have a greater influence on the extracted phasor features and, consequently, on the classification performance.

While Z-score normalization operates in the spectral domain (reflectance values) prior to phasor transformation, the STD was computed from the phasor values and used as an independent feature alongside the corresponding average phasor values in the classification stage. In this way, the STD captures the dispersion of the phasor distribution, providing complementary information to the mean representation.

**Figure 7:**
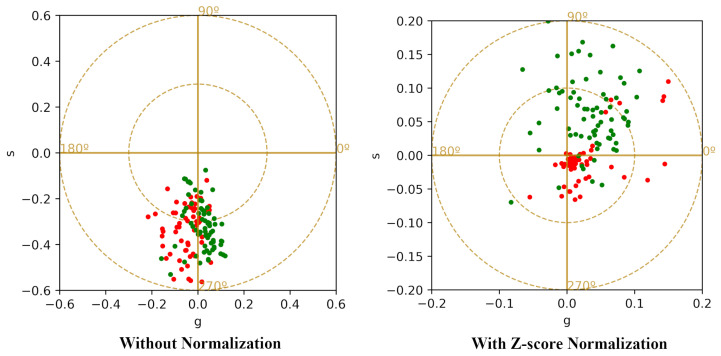
Effect of Z-score normalization on average phasor values using the first harmonic. (**Left**): Average phasor values without normalization; (**Right**): Average phasor values after applying Z-score normalization. The red points, represent diseased cases, and the green points, correspond to healthy ones.

Because spectral normalization and phasor-domain statistical descriptors affect different stages of the processing pipeline, we evaluated their impact separately and in combination. The classification results for these different scenarios are summarized in Table 6. The application of Z-score normalization also alters the position of the average phasor values, as illustrated in Figure 7, which compares the average phasor values with and without normalization.

For comparison, we also assessed classification performance using average values from each spectral band for both RGB-like and multispectral images.

Prior to calculating the average values, all images were again standardized using Z-score normalization to ensure a consistent representation of the data. For phasor value calculation, we selected the first harmonic, and employed the ν-SVC classifier, which was previously identified as the optimal choice. The classification results for these different feature sets are presented in Table 7, allowing for a direct comparison of the phasor analysis approach with traditional RGB and multispectral image classification methods.

Table 8 presents a statistical comparison between healthy and diseased groups for various feature representations derived from RGB-like and multispectral images, including averaged reflectance and phasor-based features. For the averaged reflectance features (Avg-RGB and Avg-MSI), which consisted of 3-dimensional and 12-dimensional vectors, respectively, we applied MANOVA directly to these vectors. In contrast, the phasor-based features (Phasor-RGB and Phasor-MSI) comprised 2D phasor values (g,s) for each retina. Notably, it is apparent that all types of analyses showed consistent differences (p<0.001) between healthy and diseased groups. It is noteworthy that prior to the phasor analysis, Z-score normalization was applied to both RGB-like and multispectral images and the first harmonic was used for phasor calculation.

### Analysis of Regions of Interest

The results presented thus far were obtained by analyzing each fundus image as a complete retinal image, using features extracted from the entire retina. To further investigate whether specific anatomical regions contribute differently to disease discrimination, we performed a region-wise analysis on the same retinal images. In this analysis, the retina was subdivided into ROIs and features were extracted and evaluated independently for each region. However, to ensure accurate analysis, we excluded certain diseased cases for each region, as some examples only affected one region (e.g., the optic disk) while sparing others. As a result, the number of diseased cases varied across regions, whereas the number of healthy cases remained consistent across all scenarios. The classification results for these various ROIs are summarized in Table 9.

The highest overall and balanced accuracies were achieved when features were extracted from the entire retina. Although certain regions, such as vessels and background, demonstrated relatively strong performance for specific metrics, none of the individual ROIs outperformed the full-retina analysis across all evaluation metrics. This suggests that discriminative information is dispersed across multiple retinal regions in the eyes analyzed, rather than being localized to a single anatomical area.

## 4. Discussion

The results indicate that phasor analysis is more effective than simple averaging in capturing diagnostically relevant spectral patterns in multispectral data. This improvement is likely due to the phasor representation’s ability to preserve spectral shape information, rather than reducing the spectrum to a single scalar value. In retinal imaging, where disease-related changes can be subtle and distributed across wavelengths, this shape-based encoding is particularly important.

The superior performance achieved with Z-score normalization highlights the sensitivity of retinal multispectral images to variability in acquisition and processing. Since retinal signals are inherently weak and susceptible to illumination non-uniformities and dark-current effects, classifiers that rely on absolute spectral values may inadvertently learn acquisition artifacts rather than physiological differences. By normalizing spectra using Z-score normalization, small spectral differences are emphasized, which may better reflect underlying tissue properties. This finding suggests that relative spectral contrast, rather than absolute reflectance, is a more reliable indicator of retinal pathology.

The prominence of the first harmonic in our phasor analysis is consistent with established practices in other imaging domains. In spectral and lifetime phasor approaches, the first Fourier harmonic is commonly used to capture the dominant features of a spectrum without requiring model fitting, and it forms the basis of phasor coordinates in many seminal implementations. This emphasis on low-frequency components is justified by the fact that biological spectra and lifetime decays tend to be smooth and dominated by broad trends rather than rapid oscillations [33]. In fact, a recent study using spectral phasor analysis of a fluorophore model system, demonstrated that the first harmonic was more sensitive to shifts in the emission peak, while higher harmonics were more sensitive to spectral width but also more prone to noise [34]. Similarly, our results indicate that including higher-order harmonic components did not improve classification performance and, in some cases, degraded it. This suggests that the disease-related information in retinal reflectance is primarily encoded in broad spectral features. Alternatively, it is possible that higher harmonics may not be effectively used by the selected classifiers here, particularly given the limited size of our dataset.

In our study, the comparison between multispectral and RGB-like representations underscores the importance of increased spectral resolution for accurate biomedical image classification, particularly when dealing with retinal images. Similar trends have been reported in dermatology, including a recent work on skin cancer lesion classification, where multispectral data outperformed RGB images despite the use of advanced DL models [35]. Moreover, systematic reviews in computational pathology suggest that hyperspectral and multispectral imaging can improve disease detection compared with traditional RGB analysis by using additional spectral information across wavelengths [36]. These findings demonstrate that the additional spectral information provided by multispectral systems can yield measurable diagnostic benefits beyond RGB representations.

Comparable conclusions have also been reported in retinal imaging. A study demonstrated that hyperspectral retinal images achieved superior performance compared with color fundus images for drusen detection, highlighting the advantage of exploiting extended spectral information in ophthalmic disease classification [37]. Consistent with these studies, our results show that although phasor analysis improved classification performance even when applied to RGB-like data, multispectral representations consistently achieved higher overall performance. Notably, while averaged reflectance features from multispectral images resulted in high specificity at the expense of sensitivity, phasor analysis applied to multispectral data provided a more balanced trade-off, as reflected by higher F1 Scores. This suggests that combining spectral richness with an appropriate feature representation is critical for effective retinal disease discrimination.

Although statistical hypothesis testing was incorporated to assess differences between harmonics, image types, and analysis strategies, the MANOVA results indicated statistically significant differences across all tested conditions. Consequently, the statistical analysis did not provide sufficient discriminatory power to identify an optimal harmonic order or processing approach, as significance was uniformly observed rather than selectively informative. Therefore, classification performance was adopted as the primary decision-making framework in this study. The classifier-based evaluation offers a more task-relevant and application-oriented assessment. The harmonic and analysis configuration yielding the highest classification performance was thus selected as optimal, as it maximizes discriminative capability rather than merely demonstrating statistical difference.

While many retinal pathologies initially affect localized regions, such as the macula or optic disk, the eyes in this study primarily represent advanced disease stages, where changes are widespread across multiple retinal locations. This extensive involvement may diminish the benefits of predefined regions, explaining why localized analysis did not outperform global retinal analysis in our dataset. Additionally, the limited sample size in this study may have also impacted the observed performance differences.

Moreover, the impact of ROI selection became particularly important after applying Z-score normalization. By masking the image to a limited ROI, the number of contributing pixels is substantially reduced, which can compromise the stability of averaged phasor features. With fewer pixels, the computed average phasor values become more susceptible to local variability and noise, increasing the risk that the estimated mean deviates from the underlying spectral distribution of the tissue. This effect is increased when normalization emphasizes relative spectral differences rather than absolute intensities. Therefore, restricting analysis to small or disease-nonspecific ROIs may unintentionally discard informative spectral patterns, leading to degraded classification performance.

Phasor analysis and multispectral data appear to outperform color and reflectance analysis, suggesting that ML algorithms may benefit from incorporating this information. However, when interpreting these results, it is essential to consider several limitations. The relatively small dataset size may limit the ability to fully usage of higher-order harmonics or more complex spectral representations. Furthermore, the reliance on traditional ML classifiers may constrain performance when integrating higher-dimensional or multimodal data. These factors highlight the need for future work exploring larger datasets and DL approaches, which may be better equipped to handle the increased complexity introduced by multimodal imaging, such as combined multispectral data and Optical Coherence Tomography (OCT) analysis. Future research will also prioritize expanding the dataset to include a broader range of disease stages, enabling a more comprehensive evaluation of the potential benefits of ROI-based analysis. In particular, in eyes with diseases at early stages, where only a specific region is affected, this could serve as the basis for new screening tools.

## Figures and Tables

**Table 1:** Summary of disease cases by severity.

Disease	Mild Cases	Moderate Cases	Severe Cases	Total Cases
AMD	0	3	13	16
Drusen	0	6	2	8
PVD	2	2	2	6
Epiretinal membrane	0	1	2	3
Pathological myopia	0	0	2	2
Other conditions	3	8	14	25

**Table 2:** Refractive status (sphere, cylinder), and Best-Corrected Visual Acuity (BCVA) across all subjects in the study, providing an overview of the dataset’s clinical characteristics (Mean ± STD [range]).

	Healthy Cases	Diseased Cases
Sphere (D)	0.43 ± 1.76 [−8.0, 8.0]	−0.30 ± 2.46 [−11.0, 5.0]
Cylinder (D)	−0.33 ± 0.36 [−2.0, 0.0]	−0.81 ± 1.05 [−7.0, 0.0]
BCVA	1.01 ± 0.17 [0.5, 1.5]	0.64 ± 0.32 [0.0, 1.0]

**Table 3:** Classification performance of the 4 ML algorithms: Nearest Centroid (NC), Gaussian Naive Bayes (GNB), Support Vector Machine (SVM), and ν-Support Vector Classifier (ν-SVC) over phasor values obtained from multispectral images using the first harmonic (OA: Overall Accuracy; BA: Balanced Accuracy).

Classifier	OA (%)	BA (%)	Specificity	Sensitivity	Precision	F1 Score
NC	69	69	0.75	0.62	0.69	0.65
GNB	66	66	0.78	0.54	0.69	0.59
SVM	70	70	0.80	0.61	0.72	0.65
ν-SVC	74	74	0.77	0.70	0.72	0.71

**Table 4:** Classification performance using different harmonics of multispectral images obtained with the ν-SVC classifier (OA: Overall Accuracy; BA: Balanced Accuracy).

Harmonic	OA (%)	BA (%)	Specificity	Sensitivity	Precision	F1 Score
1st	74	74	0.77	0.70	0.72	0.71
2nd	56	56	0.55	0.57	0.52	0.54
3rd	56	56	0.56	0.57	0.52	0.54
4th	69	69	0.66	0.72	0.65	0.68

**Table 5:** Statistical analysis of harmonics of multispectral images obtained with MANOVA.

	MANOVA	
Harmonic	Value [F-Value]	*p*
1st	0.789 [17.36]	<0.001
2nd	0.916 [5.93]	0.003
3rd	0.909 [6.48]	0.002
4th	0.730 [23.981]	<0.001

**Table 6:** Impact of Z-score normalization and STD on classification performance. Columns are labeled according to the applied normalization (R: Reflectance values without normalization; Z: Z-score normalization) and the extracted phasor features (AVG: Average values; STD: Standard deviation) (OA: Overall Accuracy; BA: Balanced Accuracy).

[Normalization]—[Phasor Features]	OA (%)	BA (%)	Specificity	Sensitivity	Precision	F1 Score
R–AVG	74	74	0.77	0.70	0.72	0.71
Z–AVG	85	85	0.87	0.83	0.84	0.83
R–AVG+STD	76	76	0.78	0.73	0.75	0.73
Z–AVG+STD	48	48	0.45	0.51	0.34	0.39

**Table 7:** Comparison of classification performance using different image features and analysis types. Avg-MSI and Avg-RGB denote reflectance-based baseline features obtained by averaging spectral bands of multispectral (12 bands) and RGB-like (3 bands) images, respectively. Phasor-MSI and Phasor-RGB denote phasor-based features extracted from multispectral images and from RGB-like images simulated by combining 3 multispectral bands centered at 471, 595, and 732 nm, corresponding to blue, green, and red channels (OA: Overall Accuracy; BA: Balanced Accuracy).

Analysis Type	OA (%)	BA (%)	Specificity	Sensitivity	Precision	F1 Score
Avg-RGB	76	75	0.93	0.57	0.89	0.67
Avg-MSI	80	79	0.98	0.60	0.96	0.73
Phasor-RGB	83	83	0.78	0.89	0.77	0.82
Phasor-MSI	85	85	0.87	0.83	0.84	0.87

**Table 8:** Statistical comparison between healthy and diseased groups using averaged and phasor-based features extracted from RGB-like (3 bands) and multispectral images (12 bands). Group differences were evaluated using MANOVA.

	MANOVA	
Analysis Type	Value [F-Value]	*p*
Avg-RGB	0.483 [45.99]	<0.001
Avg-MSI	0.353 [18.33]	<0.001
Phasor-RGB	0.727 [24.43]	<0.001
Phasor-MSI	0.650 [35.03]	<0.001

**Table 9:** Comparison of classification performance using the entire retina and different Region of Interest (ROIs) (OA: Overall Accuracy; BA: Balanced Accuracy).

ROIs (# Diseased Cases)	OA (%)	BA (%)	Specificity	Sensitivity	Precision	F1 Score
Entire Retina (60)	85	85	0.87	0.83	0.84	0.83
Macula (49)	50	49	0.58	0.41	0.39	0.39
Vessels (43)	78	78	0.82	0.74	0.73	0.72
Optic Disk (42)	65	63	0.73	0.54	0.55	0.54
Background (46)	82	84	0.77	0.90	0.70	0.78

## Data Availability

The original contributions presented in this study are included in the article/Supplementary Materials. Further inquiries can be directed to the corresponding author.

## Supplementary Materials

The following supporting information can be downloaded at: https://www.mdpi.com/article/10.3390/s26031021/s1, Figure S1: Representative multispectral fundus images of high-myopia subjects from the healthy control group. Two healthy control subjects with high myopia but no pathological retinal findings are shown. Images are displayed across 865 nm wavelength. In these cases, no pathological features are observed, and overall reflectance patterns remain within the range observed for the healthy cohort. Figure S2: Complete 12-channel multispectral fundus image stacks for representative diseased retina. Each retina reflectance is corresponding to a specific wavelength that is written on each image. Figure S3: Complete 12-channel multispectral fundus image stacks for representative healthy retina. Each retina reflectance is corresponding to a specific wavelength that is written on each image.

## Abbreviations

The following abbreviations are used in this manuscript:

AI	Artificial Intelligence
AMD	Age-related Macular Degeneration
CUV	Vision University Center
DL	Deep Learning
DR	Diabetic Retinopathy
FFT	Fast Fourier Transform
GNB	Gaussian Naive Bayes
IMO	Instituto de Microcirugia Ocular
ML	Machine Learning
NC	Nearest Centroid
NIR	Near Infra-red
OCT	Optical Coherence Tomography
ROI	Region of Interest
SNR	Signal-to-Noise Ratio
STD	Standard Deviation
SVC	Support Vector Classifier
SVM	Support Vector Machine
VIS	Visible
