# Supplementary material for: Comprehensive and Region-Specific Retinal Health Assessment Using Phasor Analysis of Multispectral Images and Machine Learning
Source: Sensors (Basel). 2026 Feb 4;26(3):1021. doi: 10.3390/s26031021 (PMC12900091; doi:10.3390/s26031021)
Supplement: Supplementary file 1 [file sensors-26-01021-s001.zip › sensors-4091576-supplementary.pdf]

Supplementary Material for Comprehensive and Region-Specific Retinal Health Assessment using Phasor Analysis of Multispectral Images and Machine Learning

Armin Eskandarinasab* , Laura Rey-Barroso , Francisco J. Burgos-Fernández , and Meritxell Vilaseca*

Center for Sensors, Instruments and Systems Development (CD6), Universitat Politècnica de Catalunya (UPC),

Rambla Sant Nebridi 10, Terrassa, Barcelona, 08222, Spain

Introduction

This supplementary document presents additional figures that support and expand upon the results in the main manuscript. The included figures provide a deeper examination of the dataset images and their conditions.

High Myopia Cases

This supplementary material provides representative examples of high-myopia cases included in the healthy control group. These subjects were carefully reviewed to exclude pathological findings. The figures illustrate the multispectral fundus appearance of two cases, demonstrating that despite having high myopia levels, these cases are non-pathological.

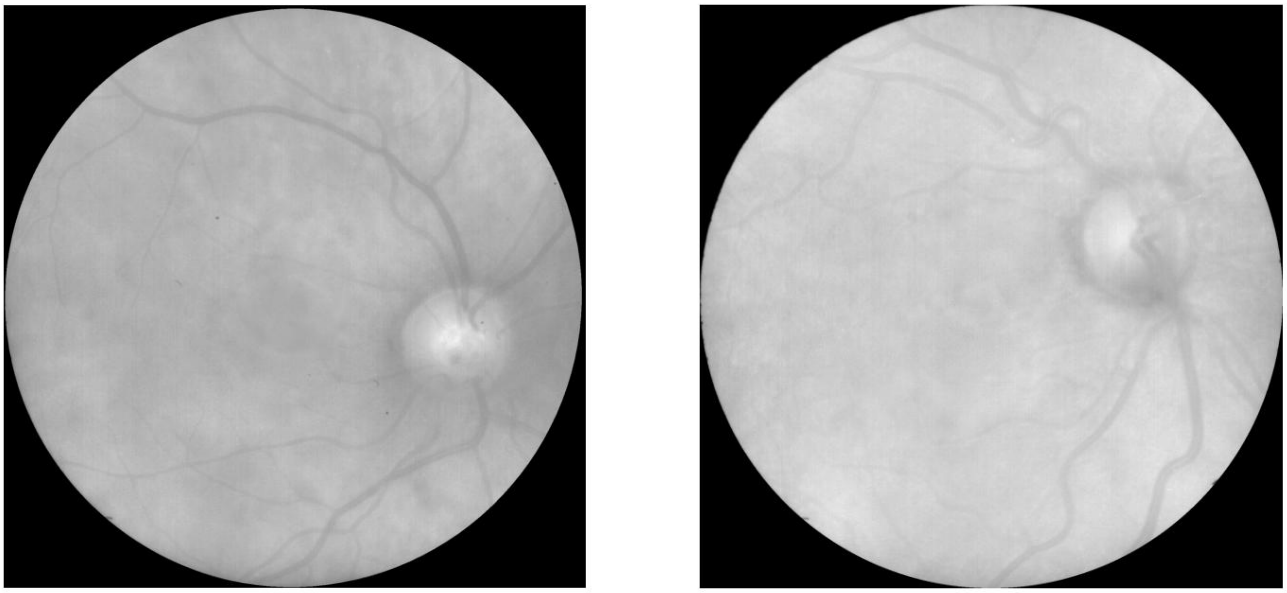

Figure S1. Representative multispectral fundus images of high-myopia subjects from the healthy control group. Two healthy control subjects with high myopia but no pathological retinal findings are shown. Images are displayed across 865nm wavelength. In these cases, no pathological features are observed, and overall reflectance patterns remain within the range observed for the healthy cohort.

Visualization of Multispectral Bands

This supplementary material provides comprehensive multispectral visualizations to complement Figures 3 and 4 of the main manuscript. Specifically, full 12-channel image stacks are shown for one representative diseased and one representative healthy retina. These visualizations are intended to help readers better understand spectral variations across wavelengths.

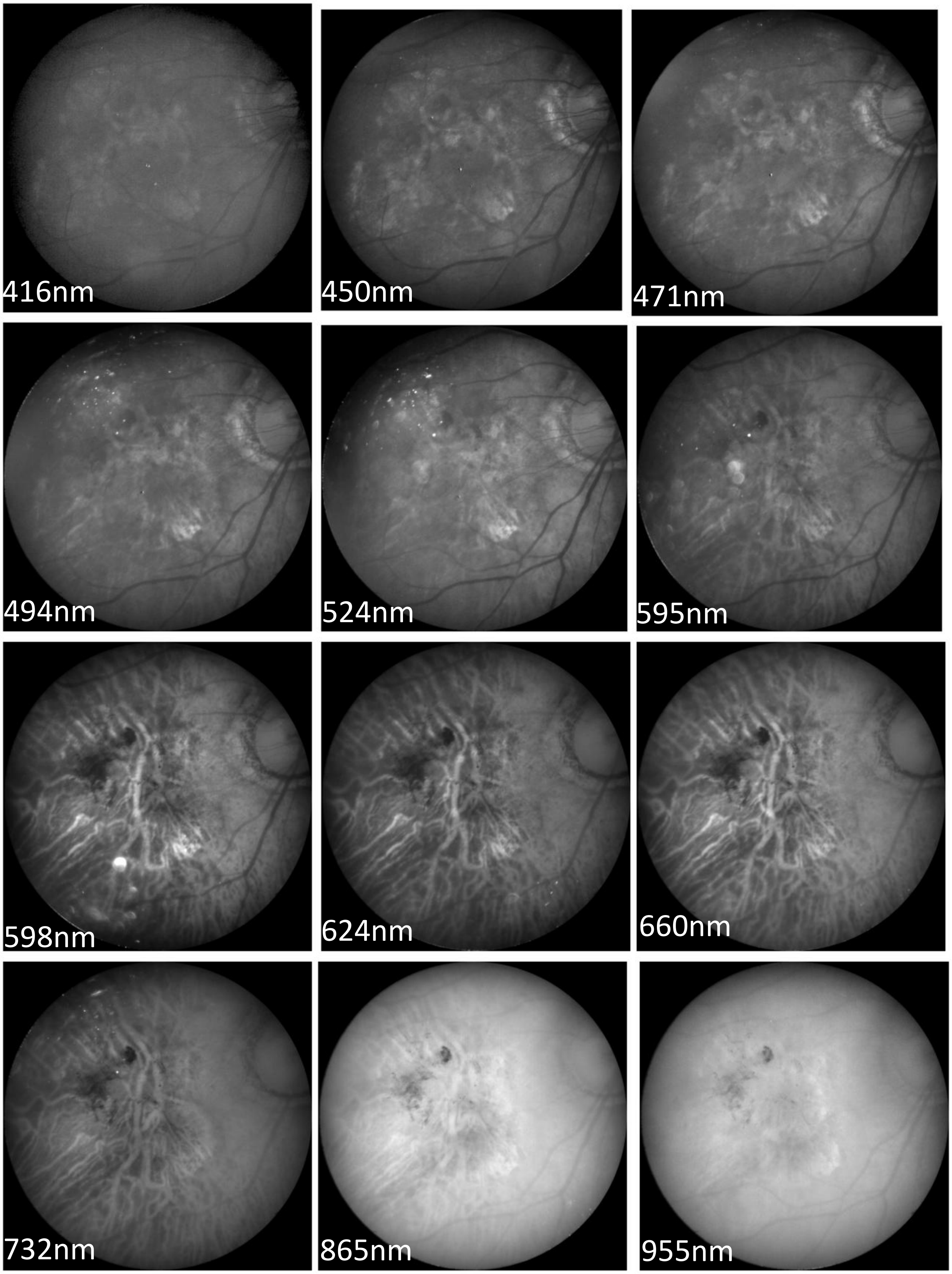

Figure S2. Complete 12-channel multispectral fundus image stacks for representative diseased retina. Each retina reflectance is corresponding to a specific wavelength that is written on each image.

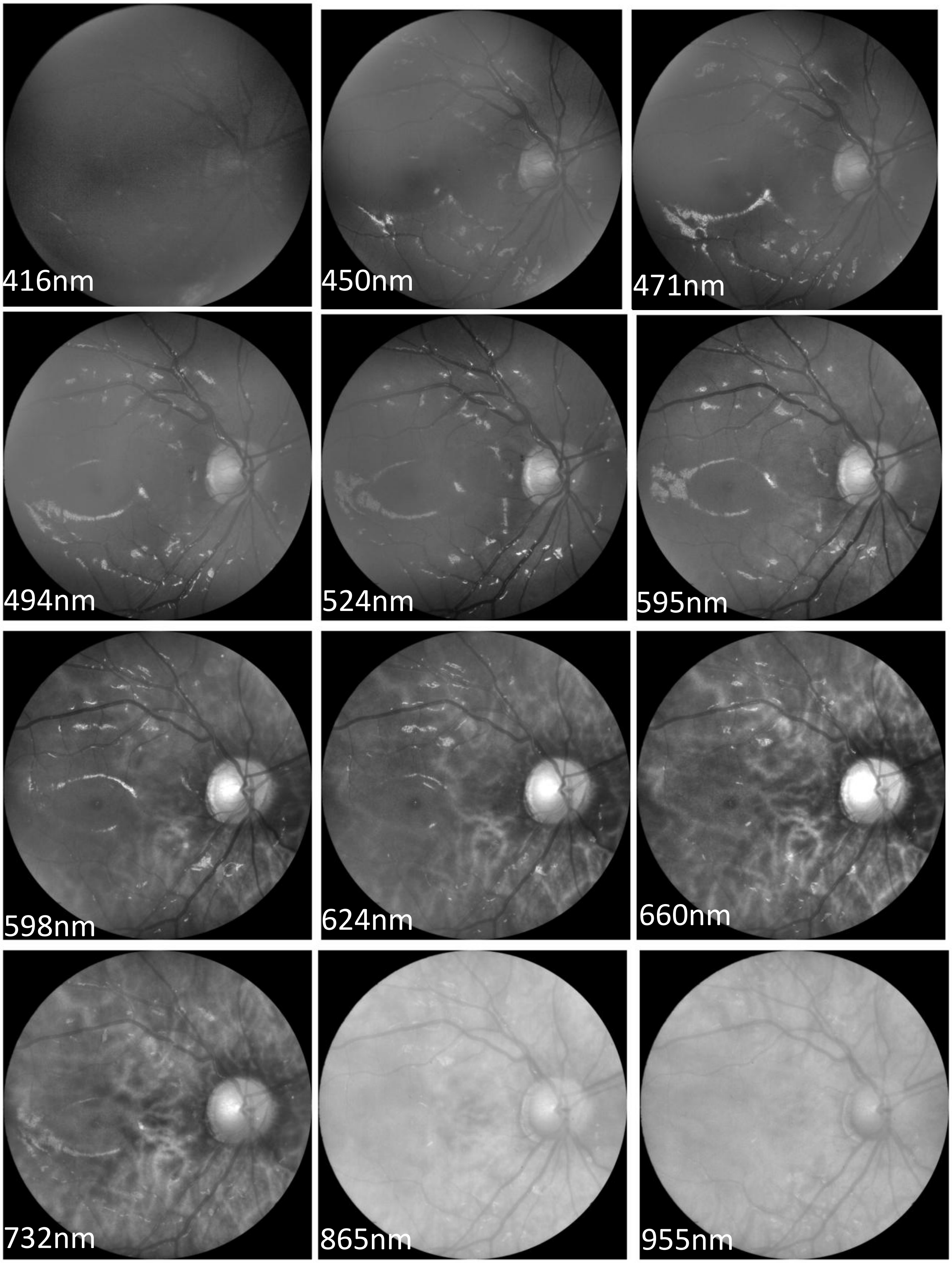

Figure S3. Complete 12-channel multispectral fundus image stacks for representative healthy retina. Each retina reflectance is corresponding to a specific wavelength that is written on each image.
